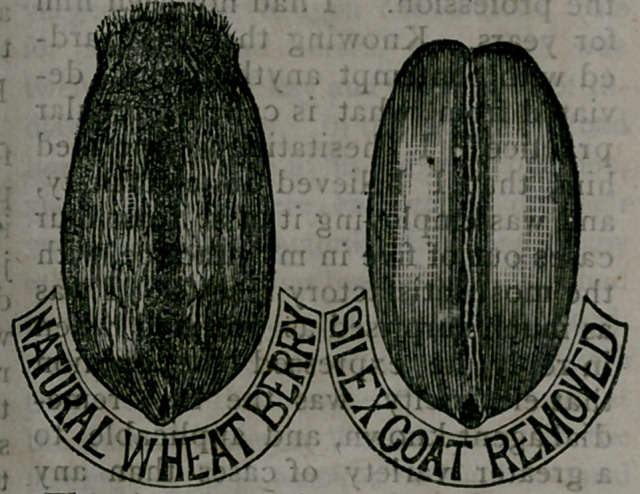# Health Food Company

**Published:** 1875-09

**Authors:** 


### THE HEALTH FOOD COMPANY.

THE name clearly expresses the business of this Company. Any article of food useful for invalids, or of special value as a wholesome diet for general use, may be regarded as "in their line." But it must not be inferred that they sell every dietetic nostrum advertised in the newspapers. The articles which they introduce to the public are perfectly pure and are very carefully prepared. Their office is at 137 Eighth Street, New York.

Their excellent Granulated Wheat Biscuit are now widely known, and their Sea Water Biscuit are just being introduced. They now present another valuable preparation which they call "Cerealine."

*Cerealine*, is a new and most excellent preparation of wheat. It is that portion of the kernel found next within the outer coating, and surrounding the starchy part, which latter is the fine flour. The Cerealine is rich in gluten and is the nutriment required for the growth and repair of muscle. It contains the lime needed for the bones and teeth, and the Phosphorus which physiologists now regard as the great food of the brain.

From this *Cerealine* a very palatable mush is made, which may be eaten warm or cold, with cream or syrup or any other dressing. It is also most excellent for griddle cakes, and ought to be substituted for buck wheat. It certainly will usurp the place of that old autocrat of the breakfast table, with those to whom buck wheat is positively injurious.

*Cerealia* is another of the wheat preparations. It is cerealine and more too. The former is simply that part of the grain containing the glutinous and mineral constituents, while the Cerealia is the entire kernel and includes the starchy portions or that which is commonly used only in the form of fine flour. It is a complete and perfect food. It may be used in any of the various ways in which Rice or Hominy or Tapioca is cooked and is more nutritious than either of these popular articles. We have just said that the *Cerealia* is the entire grain of the wheat. It *is* all of the grain which has any value as food. In this as in the *Cerealine* and in all of these preparations the outer coat of the wheat is removed. This outer coat is composed of woody substance and silex or flint. It is enveloped in a kind of *husk* formed of stiff hairs or bristles, which afford a receptacle for filth and the eggs of insects.

The accompanying cut represents a grain of wheat with the outer coating, bristles and all, and also one from which it has been removed.

Now this external coat which we may most appropriately call the husk, is useless as food. It is positively injurious from its irritating qualities. In the removal of this external and injurious part of the wheat consists the improvements in the manufacture of the *Cerealine*, *Cerealia* and other of these nice preparations, over the common articles of human food made from the grain. Persons of weak digestion who cannot use oatmeal, and graham flour or wheaten grits because they are coarse and irritating, will find *Cerealine* and *Cerealia*, free from those objections, easily digested and highly nutritious.

## Figures and Tables

**Figure f1:**